# Speech Coding in the Brain: Representation of Vowel Formants by Midbrain Neurons Tuned to Sound Fluctuations^1^,^2^,^3^

**DOI:** 10.1523/ENEURO.0004-15.2015

**Published:** 2015-07-20

**Authors:** Laurel H. Carney, Tianhao Li, Joyce M. McDonough

**Affiliations:** 1 Departments of Biomedical Engineering, and Neurobiology & Anatomy, University of Rochester, Rochester, New York 14642; 2 Department of Linguistics, University of Rochester, Rochester, New York 14627-0096

**Keywords:** auditory nerve, computational model, midbrain, modulation tuning, speech coding

## Abstract

Current models for neural coding of vowels are typically based on linear descriptions of the auditory periphery, and fail at high sound levels and in background noise. These models rely on either auditory nerve discharge rates or phase locking to temporal fine structure. However, both discharge rates and phase locking saturate at moderate to high sound levels, and phase locking is degraded in the CNS at middle to high frequencies. The fact that speech intelligibility is robust over a wide range of sound levels is problematic for codes that deteriorate as the sound level increases. Additionally, a successful neural code must function for speech in background noise at levels that are tolerated by listeners. The model presented here resolves these problems, and incorporates several key response properties of the nonlinear auditory periphery, including saturation, synchrony capture, and phase locking to both fine structure and envelope temporal features. The model also includes the properties of the auditory midbrain, where discharge rates are tuned to amplitude fluctuation rates. The nonlinear peripheral response features create contrasts in the amplitudes of low-frequency neural rate fluctuations across the population. These patterns of fluctuations result in a response profile in the midbrain that encodes vowel formants over a wide range of levels and in background noise. The hypothesized code is supported by electrophysiological recordings from the inferior colliculus of awake rabbits. This model provides information for understanding the structure of cross-linguistic vowel spaces, and suggests strategies for automatic formant detection and speech enhancement for listeners with hearing loss.

## Significance Statement

Encoding of speech sounds is the most important function of the human auditory system. Current models for neural coding of speech fail over the range of sound levels encountered in daily life and in background noise. The acoustic structure of vowels and the properties of auditory midbrain neurons that are tuned to low-frequency amplitude fluctuations suggest a neural code for the spectral peaks (called formants) that identify vowels. The proposed neural code for speech sounds is the first that is robust over a wide range of sound levels and in background noise. These results address classic problems in auditory neuroscience and linguistics, and suggest novel strategies for auditory prosthetics, automatic speech recognition, and speech enhancement for hearing aids and telephones.

## Introduction

Vowels carry a heavy functional load in all languages, especially in running speech and discourse. How vowels are encoded by the nervous system across the range of sound levels important for vocal communication is unknown. The acoustic signature of vowels includes periodicity at the fundamental frequency (F0, or voice pitch); the harmonics of F0; and formants, the amplitude bands in the spectrum that characterize vowel contrasts (Fant, 1960). The first two formants are most important for vowel identification. Studies of auditory nerve (AN) speech coding typically focus on response rates or temporal synchrony at frequencies to which a fiber is most sensitive (Sachs and Young, 1979; Young and Sachs, 1979; Delgutte and Kiang, 1984; Schilling et al., 1998). These codes are adequate for low-level speech sounds in quiet, but they fail for moderate-to-high sound levels and in background noise. Vowels also induce systematic changes in the amplitude of F0-related fluctuations in AN responses. The vowel-coding hypothesis tested here focuses on the F0-related neural fluctuations and on contrasts in their amplitudes across neurons tuned to different frequencies.

**Figure 1:**
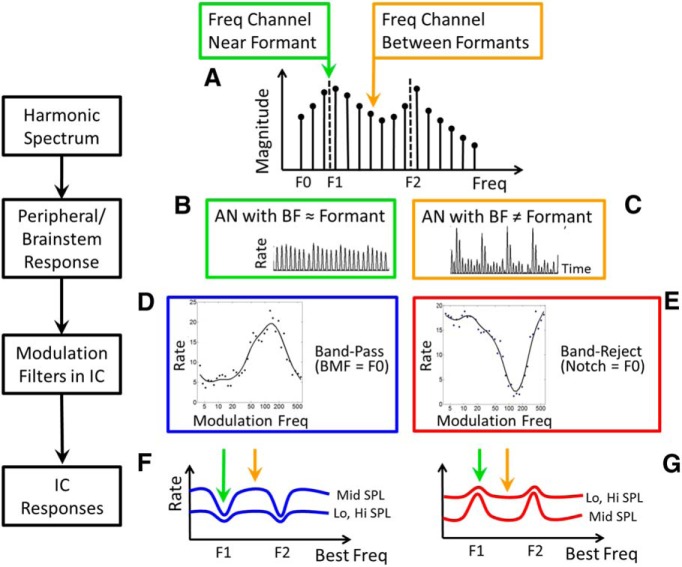
Schematic illustration of vowel-coding hypothesis. The left-hand column labels the key stages in the coding scheme. ***A***, Vowel spectrum consisting of harmonics of F0, shaped by the spectral envelope. ***B***, Responses of AN fibers tuned near formants have relatively small pitch-related rate fluctuations. These responses are dominated by a single harmonic in the stimulus, referred to as synchrony capture. ***C***, Fibers tuned between formants have strong rate fluctuations at F0 (Delgutte and Kiang, 1984). ***D***, Example of a bandpass MTF from rabbit IC with a BMF near F0 for a typical male human speaker. ***E***, Example band-reject MTF with a notch near a typical F0. ***F***, Bandpass midbrain neurons have reduced rates in frequency channels with weak fluctuations (green arrow) and increased rates in channels with strong fluctuations (see ***C***, orange arrow); thus dips in the rate profile of bandpass neurons encode F1 and F2. ***G***, The profile of rates across a population of band-reject neurons has peaks at F1 and F2, because band-reject neurons respond more strongly to stimuli that result in reduced neural fluctuations in their inputs (see ***B***, green arrow).

Many inferior colliculus (IC) neurons display both spectral tuning, described by a most sensitive best frequency (BF), and tuning to the frequency of sinusoidal fluctuations in amplitude, described by a best modulation frequency (BMF; Krishna and Semple, 2000; Joris et al., 2004; Nelson and Carney, 2007). Most IC neurons tuned for amplitude fluctuations have BMFs in the range of voice pitch (Langner, 1992) and are thus well suited to represent the critical acoustic features of vowels (Delgutte et al., 1998). The vowel-coding hypothesis presented here takes advantage of nonlinear properties of AN responses, including rate saturation (Sachs and Abbas, 1974; Yates, 1990; Yates et al., 1990) and synchrony capture, which is the dominance of a single stimulus frequency component on the response (Fig. 1; Young and Sachs, 1979; Deng and Geisler, 1987; Miller et al., 1997). These nonlinearities have strong effects on the rate fluctuations of AN fibers in response to vowels and provide a robust framework for encoding vowel features.

Figure 1 introduces the main features of the vowel-coding hypothesis. The harmonic structure of vowels (Fig. 1A) yields two types of periodicity that coexist in AN responses, as follows: phase locking to harmonics near the BF of the fiber; and phase locking to slow fluctuations at F0. Fibers tuned near formant peaks may be saturated, and these fibers also have sustained responses that are dominated by phase locking to a single harmonic near the BF of the fiber, which is referred to as synchrony capture. Both of these nonlinearities result in responses with relatively weak fluctuations at F0 (Fig. 1B). Fibers tuned to frequencies away from formants are not dominated by one harmonic but are influenced by the beating of multiple harmonics, resulting in strong low-frequency neural fluctuations at F0 (Fig. 1C).

The contrast in the amplitude of low-frequency rate fluctuations across the AN population is enhanced in the midbrain by the rate tuning of IC neurons to amplitude modulations, which is described by modulation transfer functions (MTFs; Fig. 1D,E). The majority of MTFs in the IC have bandpass (BP) tuning to amplitude modulations (Fig. 1D), and the rest have band-reject tuning (Fig. 1E), low-pass or high-pass tuning (Nelson and Carney, 2007), or more complex MTFs that combine excitation and inhibition (Krishna and Semple, 2000). Midbrain cells with bandpass MTFs that have maxima (i.e., BMFs) near F0 are hypothesized to display decreased rates when the BF of the cell is near a formant frequency (Fig. 1F, green arrow) because the neural inputs have weak low-frequency rate fluctuations (Fig. 1B). Cells with bandpass MTFs but with BF between formants are hypothesized to have increased rates (Fig. 1F, orange arrow) because their neural inputs have strong low-frequency fluctuations (Fig. 1C). In contrast, cells with band-reject or low-pass MTFs and minima near F0 will have increased rates when formant frequencies are near the BF (Fig. 1G, green arrow), because their neural inputs have weak low-frequency fluctuations (Fig. 1B). Band-reject or low-pass cells will have decreased rates (Fig. 1G, orange arrow) when the BF is between formants and the inputs have strong fluctuations (Fig. 1C).

The contrast across frequency in the F0-related neural fluctuations sets up a code for formants that is translated into rate profiles in the midbrain (Fig. 1F,G). This study used computational models for AN fibers and IC neurons to explore the robustness of this proposed code across a wide range of sound levels and in background noise. Examples of recordings from IC neurons in awake rabbits support the model for cells that have simple bandpass or band-reject amplitude modulation tuning.

## Materials and Methods

### Modeling

**Figure 2:**
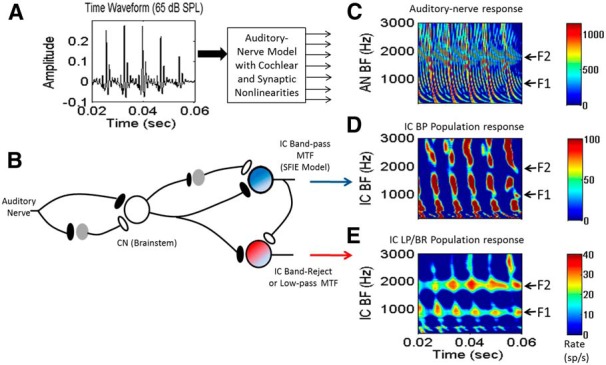
Models for modulation tuning in IC cells. ***A***, Time waveform of the vowel /æ/ (had). ***B***, The SFIE model (Nelson and Carney, 2004) for midbrain cells with BP MTFs (blue cell). An extension of the SFIE model is illustrated by the red cell, which is excited by ascending inputs and inhibited by the bandpass SFIE cell. This model cell simulates the relatively common low-pass or band-reject MTFs (see Fig. 3), and is referred to as the LPBR model. ***C***, Model AN population response (Zilany et al., 2009, 2014). ***D***, Population response of the BP IC model; BP neurons with BFs near F1 and F2 (arrows at right) have decreased responses (Fig. 1*F*). ***E***, The LPBR model has peaks in the population rate profile near F1 and F2 (Fig. 1*G*).

A phenomenological model of AN responses that includes several key nonlinearities, including rate saturation, adaptation, and synchrony capture (Zilany et al., 2009, 2014) provided the inputs to the models for two types of midbrain neurons (Fig. 2A). IC cells with BP MTFs were simulated using the same-frequency inhibition-excitation (SFIE) model (Nelson and Carney, 2004), which explains tuning for the amplitude modulation frequency by the interaction of excitatory and inhibitory inputs with different dynamics. IC cells with low-pass, band-reject (LPBR), or high-pass MTFs were simulated using an extension of the SFIE model; the LPBR model received excitatory input from the brainstem and inhibitory input from bandpass cells (Fig. 2B). Time-varying input rate functions to each model cell were convolved with α functions representing excitatory or inhibitory postsynaptic responses. The decay time constants of the α functions and the delays associated with synaptic responses were varied to produce MTFs tuned to different amplitude modulation frequencies (Nelson and Carney, 2004).

The parameter values for the cochlear nucleus (CN)/brainstem neurons (Fig. 2) were fixed for all simulations. These parameters were the time constants of the excitatory and inhibitory α functions, τ_CNex_ and τ_CNinh_; the delay of the inhibitory α function with respect to the excitatory α function, D_CNinh_; and amplitude scalars for the excitatory and inhibitory α functions, A_CNex_ and A_CNinh_ (for parameter values, see Table 1). These parameters resulted in “generic” cochlear nucleus or other brainstem neurons that project to the IC. In general, many types of CN/brainstem neurons have an increased synchrony to the stimulus envelope and a weak rate tuning to the amplitude modulation rate (Frisina et al., 1990; Grothe et al., 1997; Joris and Yin, 1998; Gai and Carney, 2008; for review, see Joris et al., 2004). The model CN/brainstem cells do not have significantly greater contrast in the rate versus BF profiles in response to vowels than do AN fibers, thus this stage of the model is not an attempt to simulate the CN “chopper” neurons described by Blackburn and Sachs (1990), which have weaker temporal representations of vowels than AN fibers or other CN response types but more robust rate versus BF profiles. The detailed response properties and connections of different types of CN and other brainstem neurons were not included in the simulations here.

**Table 1:** Model parameters

CN/brainstem parameters (all simulations)			
τ_ex_ (ms)	0.5		
τ_inh_ (ms)	2		
*D* (ms)	1		
A_ex_	1.5		
A_inh_	0.9		
IC model parameters			
Bandpass model	Fig. 3A	Fig. 3B	Fig. 3C
τ_BPex_ (ms)	2	0.7	5
τ_BPinh_ (ms)	6	0.7	10
D_BP_ (ms)	2	1.4	2
A_BPex_	2	3	6
A_BPinh_	2.2	4.2	6.6
Low-pass/band-reject model	Fig. 3A	Fig. 3B	Fig. 3C
τ_LPBRex_ (ms)	2	0.7	5
τ_LPBRinh_ (ms)	5	5	5
D_LPBR_ (ms)	0.7	0.7	0.7
A_LPBRex_	0.6	1	0.6
A_LPBRinh_	2	2	2

**Figure 3:**
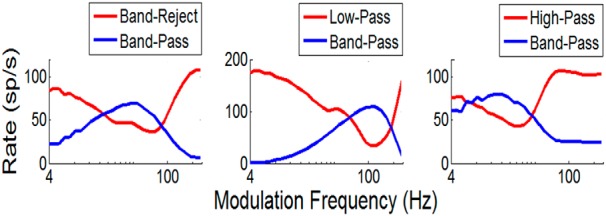
***A–C***, Three bandpass MTFs (blue, as in Fig. 2) with mid-frequency (***A***), high-frequency (***B***), and low-frequency (***C***) BMFs. MTFs for three model cells (red, as in Fig. 2) that are inhibited by the bandpass cells explain three other MTF types in the IC: the more common band-reject (***A***) and low-pass (***B***) MTFs, as well as the less common high-pass MTF (***C***). Model parameters are in Table 1.

A single set of parameters was specified for the CN/brainstem level of the model. Three sets of parameters were used for the IC models, illustrated in Figure 3, which had BMFs of 45 Hz (left), 125 Hz (middle), and 16 Hz (right). The model parameters for other figures were the same as those for Figure 3B, which had a BMF near F0 for most of the vowels used as stimuli.

Parameter values for model IC neurons are provided in Table 1. For model bandpass neurons, there were the following four parameters: the time constant of the excitatory α function, τ_BPex_; the time constant of the inhibitory α function, τ_BPinh_; the delay of the inhibition with respect to the excitation, D_BPinh_; and amplitude scalars for the excitatory and inhibitory inputs, A_BPex_ and A_BPinh_. These parameters were based on example model neurons with a range of BMFs in the study by Nelson and Carney (2004). Model band-reject, low-pass, and high-pass neurons (Fig. 2B) were described by the bandpass model parameters, plus the time constant of the excitatory α function, τ_LPBRex_; the time constant of the inhibitory α function, τ_LPBRinh_; the delay of the inhibition with respect to excitation, D_LPBRinh_; and amplitude scalars for the excitatory and inhibitory inputs, A_LPBRex_ and A_LPBRinh_.

For all models, the synaptic output signal from the auditory nerve model (which has units of spikes per second) was convolved with excitatory and inhibitory postsynaptic potentials for the CN/brainstem model. These potentials were modeled by α functions, each described by a time constant, and each normalized to have unit area before scaling the amplitudes with the coefficients described above. The model cell output was computed by subtracting the inhibitory signal from the excitatory potential and then half-wave rectifying. This model output signal was then convolved with the appropriate α function to provide the input to the next model cell, and excitatory and inhibitory signals were summed and half-wave rectified to compute the model IC response.

The basic properties of the model responses to the vowel /æ/ (in “had”) are illustrated in Figure 2C as a function of time for a population of neurons tuned to a range of BFs (the frequency that elicits the strongest response). As illustrated schematically in Figure 1, the model AN fibers (Fig. 2C) tuned near formant frequencies (arrows at right) have strong response rates with relatively small F0-related fluctuations, and those tuned to intermediate frequencies have strong fluctuations in rate associated with each pitch period. A population of model BP cells with the MTF tuned to F0 and a range of BFs is illustrated in Figure 2D. BP cells with BFs tuned to formant frequencies have weak responses compared to those tuned to intermediate frequencies, where the strong F0-related fluctuations elicit strong responses (Figs. 1C, 2D). In contrast, model LPBR cells (Fig. 2E) with a notch in the MTF near F0 (Fig. 1E) respond best when the BF is tuned near a formant frequency; these cells respond weakly to the strong F0-related fluctuations on their inputs (Fig. 1C), and are more effectively driven by the weaker modulations provided by the auditory periphery at the formant frequencies (Fig. 1B).

Figure 3 illustrates MTFs for three model BP neurons (blue curves) tuned to different amplitude modulation rates. Each of these model BP neurons provided an inhibitory input to an LPBR neuron, yielding the other set of MTFs (red curves). The shapes of these MTFs are characteristic of MTF types referred to as band reject, low pass, and high pass (Krishna and Semple, 2000; Nelson and Carney, 2007). This relatively simple model for modulation frequency tuning explains several of the MTF types that are encountered in the IC. Other IC cells have more complex MTFs, consisting of combinations of excitatory and inhibitory regions (Krishna and Semple, 2000). Further extensions of this modeling approach will be required to describe those MTFs.

### Physiological methods

All animal procedures were performed in accordance with the regulations of the University of Rochester animal care committee. Recordings were made in the central nucleus of the IC in female awake rabbits using implanted tetrodes advanced through the IC with a head-mounted microdrive (5-Drive, Neuralynx). Tetrodes were constructed by twisting four strands of 12- or 18-µm-diameter epoxy-coated platinum iridium wire. Action potentials were sorted off-line based on spike shapes (Schwarz et al., 2012). Single-unit recordings were identified based on a criterion of <2% for interspike intervals <1 ms and, when multiple spikes were recorded and sorted, on values <0.1 of the summed cluster separation metric (*L*_Σ_, based on a sum of the cluster isolation metric *L*_ratio_, from Schmitzer-Torbert et al., 2005; Schwarz et al., 2012).

Acoustic stimuli were created in Matlab and presented using Tucker-Davis Technologies hardware and Beyer Dynamic headphones through custom earmolds. Stimuli were calibrated using an Etymotic ER-7C probe-tube microphone. Audio frequency tuning was determined using response maps based on responses to 200 ms tones presented every 600 ms with frequencies from 0.2 to 20 kHz and levels from 10 to 70 dB SPL, presented in random order. Amplitude modulation tuning was determined using 100% modulated wideband noise (30 dB SPL spectrum) or tone carriers (70 dB SPL) near the best frequencies of the neurons. Vowel stimuli (65 dB SPL) were from the database of Hillenbrand et al. (1995). Samples were extracted from the steady-state portion of the vowel, and a Hanning window was applied to limit the duration to 200 ms. Vowel stimuli were chosen from the database based on the match of the speaker’s average F0 to the BMF of the neuron.

## Results

### Model responses

**Figure 4:**
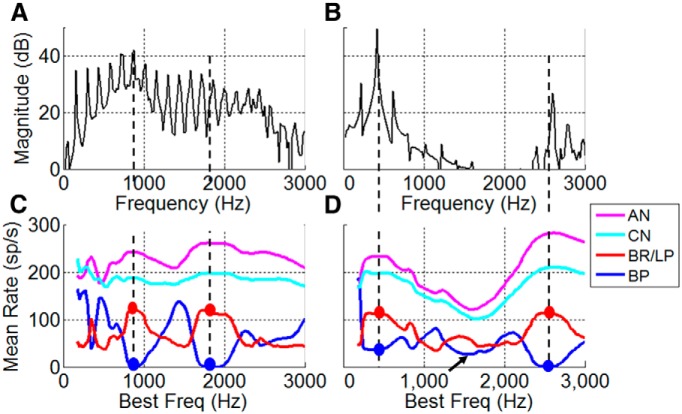
Model IC responses to vowel tokens. ***A***, ***B***, The spectra of actual vowels are as follows: /æ/ (***A***), /i/ (***B***). ***C***, ***D***, Rate profile of model cells with BP MTFs tuned to F0 (blue) has dips at formants (dots). Rate profile of LPBR model cells with minima in the MTF near F0 (red) has peaks near formants (dots). AN (magenta) and CN/brainstem (cyan) rate profiles. ***D***, Rate profile for BP cells has an ambiguous dip (arrow) for vowels with a broad spectral valley. LPBR cells (red) have relatively low rates where spectral energy is low, due to reduced excitatory inputs. Vowel levels were 65 dB SPL. Model parameters are the same as in Fig. 3*B*.

Responses of model IC cells with bandpass and band-reject MTFs to two vowel tokens (Fig. 4) illustrate the representation of formants in the average rate profiles of model IC population responses. As schematized in Fig. 1, the profile of average discharge rates for a population of model IC neurons with BP MTFs (Fig. 4C,D, blue) has minima at BFs near the vowel formants. In contrast, the rates of band-reject neurons (Fig. 4C,D, red) have peaks at the formants. The importance of the LPBR model for a robust neural code of vowel formants is illustrated in Figure 4D for the vowel /i/, which, like many vowels, has widely spaced formants. This response shows that reductions in the discharge rate of BP responses (Fig. 4D, blue) are ambiguous, as they may be due either to reduced fluctuations of AN responses tuned near formants (Fig. 1B) or to reduced spectral energy (Fig. 4D, arrow, 1500 Hz). This ambiguity is resolved by the LPBR model (Fig. 4D, red), which only responds when both sufficient energy and reduced fluctuations are present on the inputs to the model midbrain cell. The reduced fluctuations result in the disinhibition of the LPBR model by reducing the inhibitory input from the BP neuron. Note that the model LPBR population rate profile is qualitatively similar to the AN (Fig. 4C,D, magenta) and CN/brainstem (Fig. 4C,D, cyan) profiles, except that the LPBR population responses (Fig. 4C,D, red curves) have greater contrast and steeper slopes in the discharge rates across the population in comparison with the peripheral responses. The LPBR model average rate profiles differ from peripheral rate profiles in being more robust for vowels in background noise and across a wide range of sound levels (see below).

**Figure 5:**
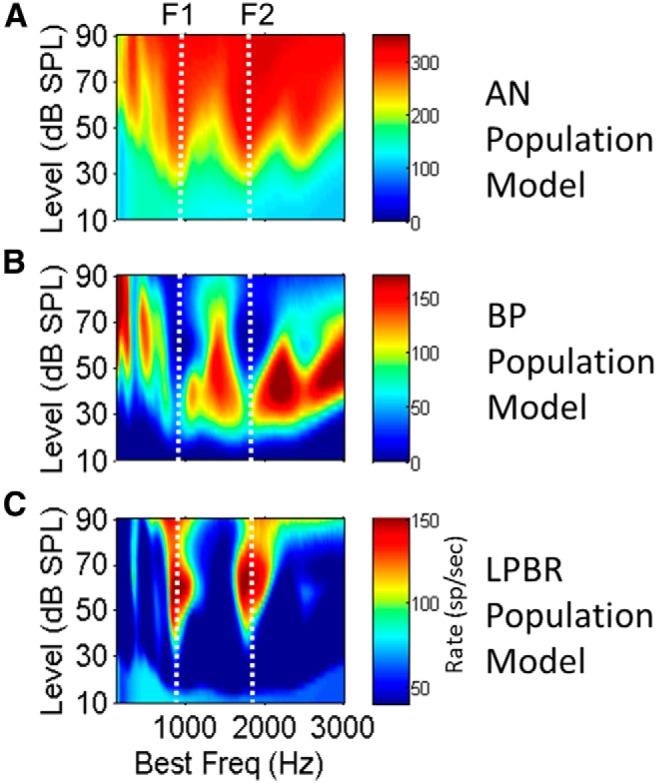
***A–C***, Population rate profiles for model AN (***A***), BP (***B***), and LPBR (***C***) cells in response to the vowel /æ/ (had) for a range of sound levels. Vertical dotted lines indicate the first two formant frequencies. ***A***, Peaks (red) in AN rates that code formants at low SPLs saturate as level increases. ***B***, Dips (blue) in the rate profile for F1 and F2 extend from ∼30 to 95 dB SPL and are strongest at conversational speech levels of 50-70 dB SPL. ***C***, LPBR model cells have peaks (red) in the rate profile at the formant frequencies; these peaks are most robust at conversational speech levels. Model parameters are the same as in Fig. 3*B*.

The midbrain vowel-coding hypothesis is robust across a wide range of SPLs (Fig. 5) because it is based on the pattern of pitch-related rate fluctuations in AN responses, as opposed to spectral energy or average rates of AN fibers. Model AN rates, shown in response to the vowel /æ/ (had), saturate at moderate-to-high sound levels, obscuring the representations of formant peaks (Fig. 5A). All model responses presented here are based on models for low-threshold high-spontaneous rate AN model fibers, which are the majority of AN fibers (Liberman, 1978). Although responses of medium- or low-spontaneous rate fibers have somewhat larger dynamic ranges and higher thresholds, the representation of formant peaks in all fiber types weakens as the sound level increases and the fibers begin to saturate.

The representations of F1 and F2 for the vowel /æ/ (had) in the average discharge rate profiles of populations of model IC cells appear as decreased rates for model BP cells tuned near the formants (Fig. 5B, vertical blue streaks) or increased rates for model LPBR cells (Fig. 5C, vertical orange streaks). The contrast in rates [e.g., the difference between peaks (Fig. 5B,C, red) and minima (Fig. 5B,C, blue)] along the frequency axis varies with SPL. This contrast is strongest for sound levels near 65 dB SPL (Fig. 5B,C; i.e., in the range of conversational speech). The wide dynamic range of the formant representation is due partly to spike rate adaptation (Dean et al., 2005, 2008; Wen et al., 2009, 2012), which increases the overall dynamic range of auditory neurons, a phenomenon largely explained by the power law synaptic adaptation included in the AN model (Zilany and Carney, 2010).

The reduction in the contrast of rates in the model responses at high levels is consistent with the phenomenon of “rollover,” the gradual decrease in speech recognition scores at levels exceeding 80 dB SPL (Studebaker et al., 1999). The addition of smaller percentages of medium- and low-spontaneous rate AN fibers to the high-spontaneous rate model population would slightly increase the model dynamic range, but the representation of formants would still roll off at the highest levels (data not shown). The high-spontaneous rate AN models were used as inputs for the IC models shown here to emphasize that the information required for the wide dynamic range of the proposed coding hypothesis is present even in this group of AN fibers, which has the smallest dynamic range.

**Figure 6:**
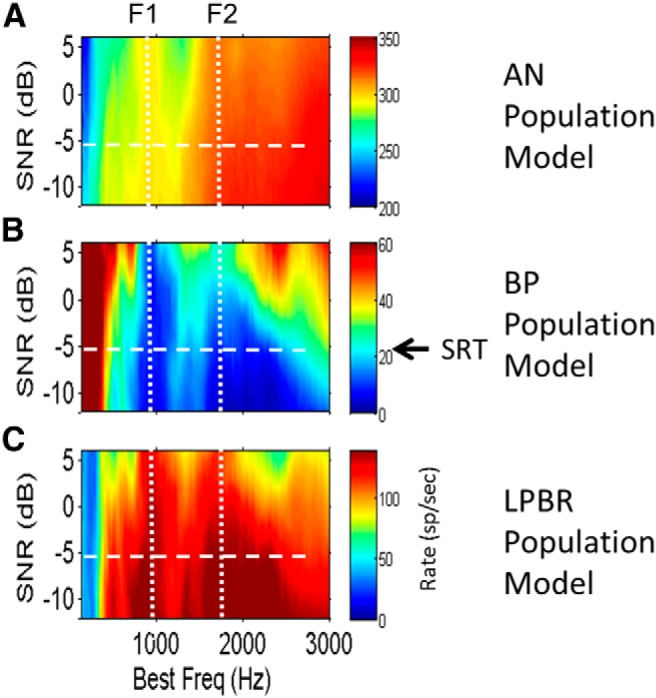
***A–C***, Population rate profiles for model AN (***A***), BP (***B***), and LPBR (***C***) cells in response to the vowel /æ/ (had) for a range of SNRs. Vowel levels were fixed at 65 dB SPL; the noise level increases toward the bottom of plots. ***A***, Saturation of AN rates by the added noise obscures representations of formant frequencies, especially in the F2 region. ***B***, Dips in the average discharge rate profile that indicate the first two formants in the BP population response deteriorate gradually as SNR decreases (toward the bottom of the plot). ***C***, Peaks in the rate profile versus SNR for model LPBR cells also deteriorate as SNR decreases. Arrow and horizontal dashed lines indicate the approximate SRT for listeners with normal hearing (Festen and Plomp, 1990). Model parameters are the same as in Fig. 3*B*.

The representation of formants in the model midbrain average discharge rate profiles is also robust in the presence of additive speech-shaped Gaussian noise across a range of signal-to-noise ratios (SNRs; Fig. 6). Figure 6A shows model AN fibers in response to the vowel /æ/ (had); as SNR decreases, the representation of the formants in the AN discharge rates deteriorates, especially in the F2 frequency region. Formant representation is much more robust in the response profiles of midbrain neurons (Fig. 6B,C). The dips in the response profiles of the model BP cells (Fig. 6B) and in the peaks in the LPBR profile (Fig. 6C) deteriorate at approximately the speech reception threshold (SRT), where human listeners have difficulty understanding speech in noise (approximately −5 dB SNR; Festen and Plomp, 1990).

### Physiological responses

The vowel-coding model was tested with recordings from IC neurons in awake rabbits in response to 12 contrastive English vowels from one human male speaker with an average F0 of 128 Hz (Hillenbrand et al., 1995). The responses of 75 neurons with BFs <4500 Hz that responded to 65 dB SPL vowel stimuli were compared to model predictions; a subset of these neurons were also studied at multiple SPLs and SNRs.

**Figure 7 F7:**
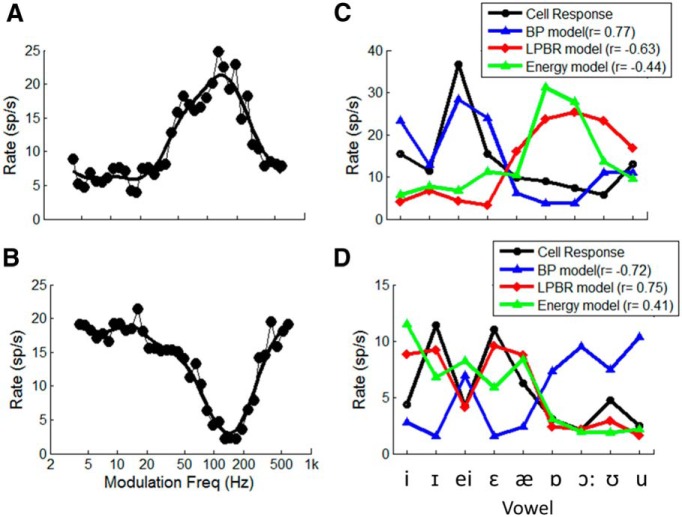
***A***, ***B***, Examples of two IC neurons in awake rabbits with bandpass MTF (BF, 1300 Hz; BMF, 130 Hz; ***A***) and band-reject MTF (BF, 2000 Hz; MTF notch at 150 Hz; ***B***). ***C***, Black, Average rate of the bandpass neuron in response to nine vowels with F0 = 148 Hz (Hillenbrand et al., 1995), 65 dB SPL. Blue, Responses of the bandpass SFIE model; red, LPBR model responses; green, energy at the output of a fourth-order gammatone filter at the BF of the cell. Mean and SD of model responses were matched to neural responses. Lines connect the symbols to emphasize patterns in the responses across this set of vowels. ***D***, Average rate of the band-reject neuron (black) to vowels with F0 = 95 Hz presented at 55 dB SPL, with LPBR model predictions (red), energy (green), and for comparison, the SFIE model response (blue). Model parameters were the same as in Fig. 3*B*.

Figure 7 illustrates responses of two neurons, one with a BF of 1100 Hz and a bandpass MTF (Fig. 7A), and the other with a BF of 2000 Hz and a band-reject MTF (Fig. 7B). Figure 7, *C* and *D*, shows the average discharge rates for these two cells in response to nine English vowels (black line), along with predictions provided by the BP SFIE (Figs. 1, 4, blue line) and LPBR (Figs. 1, 4, red line) models. For comparison, predictions based on the energy through a gammatone filter centered at the BF are also shown (Figs. 1, 4, green line). The Pearson product moment correlation coefficient between actual rates and each of the predictions is also shown.

The discharge rates of the BP cell were not explained by the stimulus energy near the BF of the neuron. For example, for the cell in Figure 7, *A* and C, the energy near the BF is greatest for the vowels /ɒ/ (in “f**a**ther”) and /ɔ:/ (in “b**aw**d”), yet the discharge rates of the neuron are low in response to these vowels. In contrast, the neuron responds strongly to /i/ (in “h**ee**d”) and /I/ (in “h**i**d”), which have relatively low energy near the BF of this neuron (Fig. 7C). The BP SFIE model, however, explains these counterintuitive responses of the BP IC neurons to vowels (Fig. 7C, blue and black lines). The responses of the BP neuron decreased when formant frequencies encroached upon the BF of the neuron (1300 Hz), as predicted by the SFIE model (Figs. 1F, 4C, blue), because of the reduced rate fluctuations in the those frequency channels. Synchrony capture and saturation of AN fibers tuned near the formant peaks result in reduced rate fluctuations in the responses of those frequency channels. Knowledge of the BF, MTF type, and BMF of the neuron allowed predictions of the vowel responses of the BP cell by the SFIE BP model.

The responses of the band-reject neuron (Fig. 7D, black) increased when formant frequencies were near the BF of the neuron (2000 Hz), as predicted by the LPBR model (Fig 7D, red). Although the responses of the band-reject neuron were positively correlated to energy near the BF (Fig. 7D, green), the LPBR model responses also reflected trends in the responses of the band-reject neuron that were not explained solely by the stimulus energy in the critical band centered at the BF.

**Figure 8 F8:**
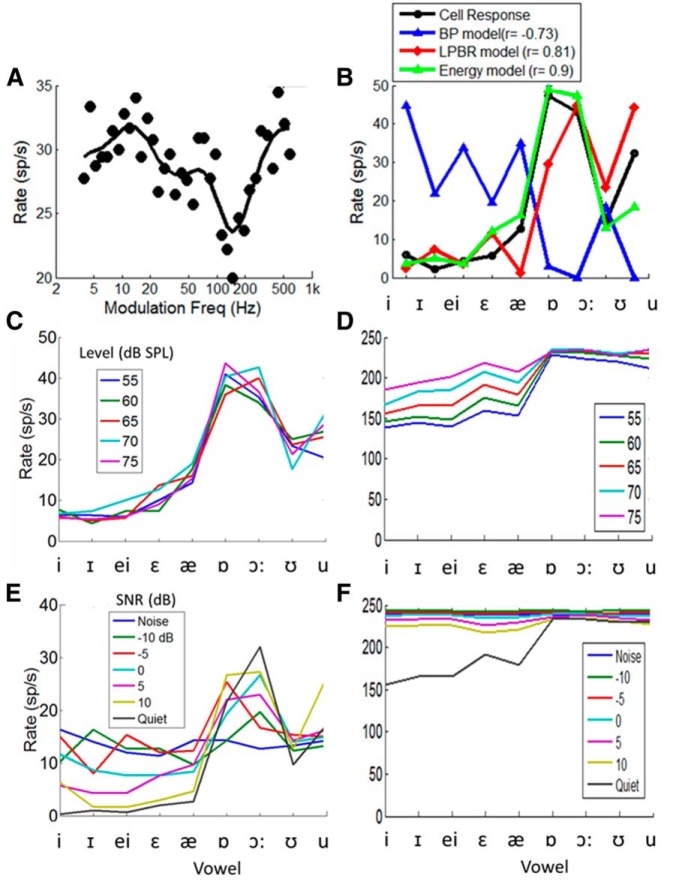
***A***, ***B***, Example of a neuron (BF, 1100 Hz) with a band-reject MTF (***A***) for which average discharge rates in response to 65 dB SPL vowels were best predicted by the LPBR model or the energy model (***B***). ***C***, ***E***, The patterns of average discharge rates for this neuron across the set of vowels were consistent across a range of SPLs (***C***) and SNRs (***E***). ***D***, ***F***, Vowel responses for a model AN fiber with the BF at 1100 Hz is shown for the same range of SPLs (***D***) and SNRs (***F***). Vowel F0 for all datasets was 95 Hz. IC Model parameters were the same as in Fig. 3*B*.

An important property of the proposed model for vowel coding is its resilience across SPL (Fig. 5) and SNR (Fig. 6). Some cells in the IC have discharge rate profiles that are similarly robust across a wide range of stimulus parameters. An example is shown in Figure 8. This neuron had a band-reject MTF (Fig. 8A), and its discharge rates in response to the set of nine vowels presented at 65 dB SPL were well predicted by the LPBR model and by the energy model (Fig. 8B). The large differences in rate across the set of vowels for this neuron facilitate comparisons of the rate profile across a range of SPLs (Fig. 8C) and SNRs (Fig. 8E). As SNR decreases, the rate profile approaches the response to 65 dB noise alone (Fig. 8E, blue), whereas at high SNRs the profile approaches the response to speech in quiet (Fig. 8E, black). For comparison, responses of a high-spontaneous rate model AN fiber with the same BF (1100 Hz) are shown for the same range of SPLs (Fig. 8D) and SNRs (Fig. 8E). The AN rates across this set of vowels gradually saturate over this range of sound levels (Fig. 8D). All of the AN responses for stimuli that included the added speech-shaped noise were saturated for the SNRs studied (Fig. 8E).

**Figure 9 F9:**
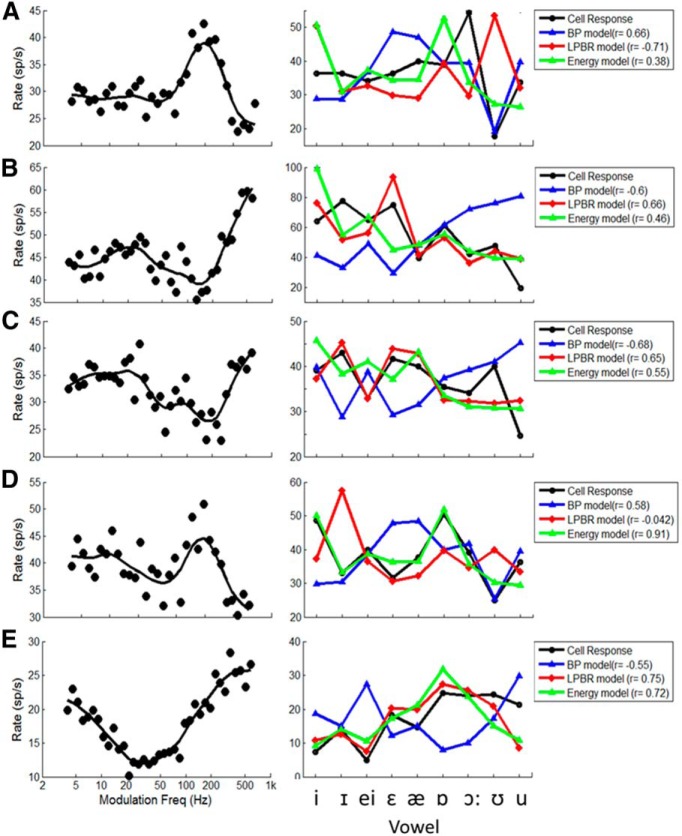
Example of five neurons with diverse MTFs (left panels) and predictions of responses to nine English vowels (right panels) at 65 dB SPL, with correlations to the model predictions in the legends. ***A–E***, BFs were 3900 Hz (***A***), 2700 Hz (***B***), 1900 Hz (***C***), 4020 Hz (***D***), and 1485 Hz (***E***). Model parameters were the same as in Fig. 3*B*.

The physiological results above demonstrate examples of IC responses with features that are consistent with the model. Of 75 neurons that responded to 65 dB vowel stimuli with F0 in the 100-130 Hz range, 62 neurons (83%) had average rates in response to a set of 12 vowels that were significantly correlated (i.e., *r* ≥ 0.57, 2 df) by at least one of the three models (BP, LPBR, or energy). Of these, 11% were best predicted by the BP model, and 42% were best predicted by the LPBR model. Note that many neurons in the IC have more complex MTFs than the simple bandpass and band-reject examples shown above. In particular, MTFs that combine excitatory and inhibitory regions at different modulation frequencies are common (Krishna and Semple, 2000), and further extension of the model is required to describe the responses of those neurons to vowels. Figure 9 illustrates diverse MTFs, vowel responses, and correlations to model predictions for five additional IC neurons. These complex MTF shapes illustrate the challenge of classifying neurons as simply bandpass or band-reject. Each of these neurons has rates that are enhanced and/or suppressed with respect to the response to the lowest modulation frequency tested. Kim et al. (2015) propose categorization of MTFs as band enhanced or band suppressed, based on comparisons to the response to an unmodulated stimulus. The examples in Figure 9 have responses that are sometimes better predicted by the BP model (Fig. 9A,D), and sometimes by the LPBR model (Fig. 9B,C,E). However, it should be noted that in some cases (Fig. 9A), the correlation between model and neural responses is strongly influenced by the responses to one or two vowels. The correlations in Figure 9 also illustrate that although the LPBR and energy model responses are often highly correlated ([Fig F7][Fig F8][Fig F9]), this is not always the case (Fig. 9A,D). In general, for the examples in Figure 9 the BP model provides better predictions of responses for neurons that have peaks in the MTF near the F0 of the stimulus, and the LPBR provides better predictions when there is a dip in the MTF near F0. Thus, it is reasonable to hypothesize that quantifying the neural fluctuations established in the periphery near the BF of a neuron, and then applying the features of the MTF at modulation frequencies relevant to the stimulus, will explain the vowel responses for cells with complex MTFs. This strategy provides a novel and general framework for understanding how complex sounds with strong fluctuations, such as voiced speech, are encoded at the level of the midbrain.

## Discussion

Previous studies of the neural representation of vowels have largely focused on the coding of spectral energy by the AN (Liljencrants and Lindblom, 1972; Delgutte and Kiang, 1984; Young, 2008; Lindblom et al., 2009). Codes based on AN average discharge rates and/or temporal fine structure are problematic because of limited dynamic range and background noise. Many AN models, especially those used in the phonetics literature, are based on linear filter banks (Lindblom, 1990; Diehl and Lindblom, 2004; Ghosh et al., 2011). The model presented here, in contrast, includes the nonlinear attributes of AN responses, including level-dependent tuning bandwidth, synchrony capture, and saturation, all of which influence the neural fluctuations in response to speech sounds that ultimately project to the IC. The coding hypothesis here focuses on the F0-related fluctuations in the AN responses and how they vary across the population. These fluctuations are important because IC neurons are tuned to both audio and modulation frequencies. This tuning provides sensitivity to the contrast in low-frequency, pitch-related fluctuations across a population of neurons with different best frequencies (Fig. 1B,C).

IC responses tuned to a range of modulation frequencies encode vowel formant frequencies across a range of pitches, capturing an essential aspect of speech (Diehl, 2008).

This model framework provides a context for understanding several general questions related to vowel systems, which exhibit universal properties that generalize across languages. For example, formant locations appear to disperse optimally within an F1–F2 space, referred to as dispersion theory (Liljencrants and Lindblom, 1972; Lindblom, 1986; Schwartz et al., 1997; Diehl and Lindblom, 2004). This dispersion results in consistencies among linguistic vowel systems in the appearance of vowel contrasts as vowel systems increase in size. Our model for neural representations of vowels thus provides a new tool for understanding how the role of the auditory system in shaping vowel systems.

This model also provides a framework to study the relative spacing of formant peaks, F1, F2, and F3, which define single vowels. The neural resolution for coding separate formants, or for merging them, depends upon both the separation of the formant peaks and the widths of the formant bands. Limits in neural resolution along the frequency axis for coding single-formant peaks would determine when nearby formants merge perceptually (Chistovich and Lublinskaya, 1979). This concept underlies focalization-dispersion theory (Schwartz et al, 1997; Becker-Kristal, 2010). In the neural model, the representation of the width of a single formant along the frequency axis (Figs. 1F, 1G, 2, 6) depends upon the width of the modulation transfer functions for these neurons (Figs. 1D,E, 4). Future studies to test the hypothesis presented here should include synthetic vowel sounds, in which the key parameters can be systematically manipulated with respect to the frequency and modulation tuning of a given neuron, as well as higher-level features such as formant spacing. These stimuli would also provide stronger statistical tests of the correlations between model and neural responses than was possible with the small set of spoken vowels used in this study.

Recent results in the cortex suggest that phonetic features are encoded in the responses of the superior temporal gyrus (Mesgarani et al., 2008; Pasley et al., 2012), but the problem of how neural maps at cortical levels are created from the acoustic stimulus remains. The results presented here suggest a framework for subcortical neural coding of phonetic features based on patterns of F0-related neural fluctuations. These patterns are established in the auditory periphery by the nonlinear response properties of inner ear mechanics and sensory transduction. Contrasts in these patterns are then enhanced by the sensitivity of midbrain neurons to fluctuation frequencies. The potential also exists for amplification of these contrasts in the thalamus and cortex by interactions between inputs from bandpass and band-reject midbrain responses. Responses of midbrain cells with complex MTFs, characterized by a combination of inhibition and excitation (Krishna and Semple, 2000), could serve as effective “edge-detectors,” further enhancing the contrasts in rate fluctuations across the neural population. In general, the combination of spectral frequency tuning and modulation frequency processing in the midbrain provides a substrate for parsing complex sounds into the features that are required for higher-level phonetic representations.

The stimuli modeled and tested in this study were limited to vowels, and the voiced structure of these sounds has a strong influence on the responses. It is interesting to consider how the properties of these neurons would influence responses to other types of speech sounds. Unvoiced vowels exist in whispered speech, and in vocoded speech, such as that used in cochlear implant simulations (Shannon et al., 1995), as well as conditioned alternates of vowels in several languages (Ladefoged and Maddieson, 1996). Unvoiced or devoiced vowels have reduced intelligibility compared to normal vowels. The model presented here would respond mainly to the energy profile in unvoiced vowels, such that formants would be coded by increased rates for neurons tuned near formants. These energy-related responses would be correlated with the representation of voiced vowels in the LPBR model, though with reduced contrast in rate as a function of frequency. Consonants represent another diverse and important set of speech sounds, sets of which (obstruents) are commonly voiceless (p, t, k, ch), and sometimes are characterized by a noisy source (e.g., fricatives; Stevens, 1998; Ladefoged, 2006). Similar to vowels, the consonants set up a pattern of neural fluctuations in the peripheral response that will ultimately drive the responses at the level of the midbrain. Future studies will extend the general approach presented here to include a larger set of speech sounds. An interesting question is how midbrain neurons with different MTFs will represent the slopes and peaks in consonant spectra, which result in nonperiodic but potentially strong fluctuations in peripheral responses.

The vowel-coding hypothesis presented here has implications for several applications related to speech processing. Accurate formant identification in the presence of substantial background noise is critical for automatic speech recognition systems, yet is difficult to achieve. The hypothesis also provides a new framework for speech enhancement algorithms for listeners with and without hearing loss. The code is substantially affected by common aspects of hearing loss, such as broadened frequency tuning in the inner ear, which distorts the representation of both the spectrum and amplitude modulations. The proposed code is also affected by changes in synchrony capture that would accompany hearing loss. Loss of synchrony capture has a profound effect on the nature of the neural fluctuations in peripheral responses, and thus on the responses of central neurons that are driven by these fluctuations. The hypothesis thus informs the development of algorithms for new hearing aids and cochlear implant speech processors that encode information in temporal envelopes.

## Synthesis

The decision was a result of the Reviewing Editor Leonard Maler and the peer reviewers coming together and discussing their recommendations until a consensus was reached. A fact-based synthesis statement explaining their decision and outlining what is needed to prepare a revision is listed below. The following reviewers agreed to reveal their identity: Eric Young, Ramnarayan Ramachandran

Both reviewers agree that this manuscript has merit in that it connects the responses properties of inferior colliculus (IC) neurons to the representation of vowels. Previous models that attempted to explain neural coding for vowels are inadequate in that they fail at high sound levels and in the presence of background noise. The model proposed in this manuscript may overcome these limitations. The basis of the model is the tuning of many IC neurons to a range of amplitude modulation frequencies. It is possible that the hypothesis advanced in this manuscript might lead to a novel and compelling explanation of vowel encoding. The reviewers have suggested many improvements to the manuscript. Many of were aimed at promoting greater clarity; in particular, for this manuscript to be accessible to as wide an audience as possible, that authors should keep jargon to a minimum and carefully explain technical terms (related to vowel perception) as they arise. One reviewer identified two problems of a more serious nature. The manuscript relies on data from only two example neurons. Clearly, there should be summary statistics from a larger IC population that will give readers some sense of how well the IC neuron population will perform in this coding task. A related point is the suggestion of a population plot of the key classes of IC neurons organized by the peak of their frequency tuning (best frequency) curves. A second critical problem is a discrepancy noted in the responses illustrated in Figure 8.

If the problems identified by the reviewers are successfully addressed, this manuscript will be an important contribution to a difficult topic and one of great relevance to language processing.

Dear Professor Maler,

Thanks you for forwarding the reviews and summary for our manuscript, "Speech Coding in the Brain: Representation of Vowel Formants by Midbrain Neurons Tuned to Sound Fluctuations." In this revised version, we have addressed all of the reviewer's concerns, as described below (in red font). Each response mentions the page of the revised manuscript that has been edited, where appropriate; the page numbers refer to the version with the changes highlighted. A "clean" copy that includes the title page will also be submitted.

The reviewer's suggestions were very helpful in revising and improving the manuscript - Many thanks to them and to you for the thorough and thoughtful suggestions.

Sincerely, The Authors

--------------------------------------------

Manuscript Instructions

- Introduction must be less than 750 words. Currently at 929 words.

> As advised by the Reviewing Editor, we consulted with the Editorial Office about the word count, and received the following reply: "You're right; the caption for figure 1 was mistakenly included in the word count for the introduction. You are clear to keep the 750 word introduction."

Thus, we have not reduced the length of the Introduction.

---------------------------------------------

Synthesis of Reviews:

Both reviewers agree that this manuscript has merit in that it connects the response properties of inferior colliculus (IC) neurons to the representation of vowels. Previous models that attempted to explain neural coding for vowels are inadequate in that they fail at high sound levels and in the presence of background noise. The model proposed in this manuscript may overcome these limitations. The basis of the model is the tuning of many IC neurons to a range of amplitude modulation frequencies. It is possible that the hypothesis advanced in this manuscript might lead to a novel and compelling explanation of vowel encoding. The reviewers have suggested many improvements to the manuscript. Many were aimed at promoting greater clarity; in particular, for this manuscript to be accessible to as wide an audience as possible, that authors should keep jargon to a minimum and carefully explain technical terms (related to vowel perception) as they arise.

>> We have edited the Introduction in an attempt to make is clearer and to reduce the use of jargon. (pages 3-5)

One reviewer identified two problems of a more serious nature. The manuscript relies on data from only two example neurons. Clearly, there should be summary statistics from a larger IC population that will give readers some sense of how well the IC neuron population will perform in this coding task. A related point is the suggestion of a population plot of the key classes of IC neurons organized by the peak of their frequency tuning (best frequency) curves.

>> This manuscript was intended as a submission for the "Theory/New Concepts, Sensory and Motor Systems" category in eNeuro; as such, an extensive physiological study was not included. The data set collected was relatively limited (75 units). We have added a brief description of the numbers of neurons with vowel responses that were statistically correlated to the two proposed model responses (Page 21). However, the design of the experiments used to test the general vowel-coding hypothesis presented here (based on sinusoidally modulated noise for the modulation transfer functions, and responses to 12 English vowels to test model predictions) provided a basic test of the hypothesis, but lacks the power to provide very meaningful statistical tests. It is difficult to sub-divide IC cells into the "key classes" based on the responses to SAM noise, for the proposed population figure. We have added text to the end of the Results section, along with a new Figure 9 to illustrate the diversity of the MTFs, with model predictions, and in the Discussion we describe these issues and pointing to future studies that will be needed to pursue them, e.g. the use of synthetic stimuli that can be varied systematically with respect to a neuron's BF and BMF. (page 25).

A second critical problem is a discrepancy noted in the responses illustrated in Figure 8.

>> There was an error in the axis of one panel in Figure 8 - this has been fixed. Thanks.

If the problems identified by the reviewers are successfully addressed, this manuscript will be an important contribution to a difficult topic and one of great relevance to language processing.

First reviewer - only requires Editorial review

This manuscript proposes a new model of auditory spectral representation by neurons in midbrain. It is a very creative idea based solidly in data and seems likely to advance the understanding of auditory stimulus representations significantly.

GENERAL COMMENTS

This manuscript investigates a clever idea in which well-known properties of neurons in IC can be used to explain the conversion of temporal representations to rate representations for vowels and vowel-like stimuli (the model in Fig. 1). A model of this process is presented and some IC data consistent with the model are shown. This is an excellent paper that proposes what may be a revolutionary way of thinking about coding of complex stimuli in the auditory system. It is clearly written and contains just enough data to make it believable.

One suggestion is that work on the vowel perception in the cochlear nucleus is not considered here, in discussing the results. The "chopper" neurons may be particularly important. Blackburn and Sachs (1990) showed reasonably robust (with sound level) representations of vowel spectra in choppers, certainly more robust than in AN fibers. Keilson et al (1997) showed a representation of the spectra of double-vowel stimuli in which the periodicity of the vowels was preserved in the responses, maybe providing a basis for the results shown here? Are the responses of the first-stage EI cells (brainstem in Fig. 2B) consistent with these cochlear nucleus data?

-The cochlear nucleus/brainstem responses included in our model are more 'generic' than the chopper responses reported in these studies. In this model, the CN stage increases synchrony to the envelope, and weak tuning to AM (see Nelson &amp; Carney, 2004), but the CN model does not enhance the rate representation significantly in comparison to that in the auditory nerve. We have added the mean rates for the model CN responses to Figure 4, and point out this issue in the Methods, when the model is introduced. (Bottom of page 7 through top of page 8).

The introduction is a little jargony and will only be understood by those experienced in the auditory periphery literature. Synchrony capture is particularly important to the working of the model. Perhaps this can be better explained by referring to Fig. 1B in the introduction? I suggest you have a colleague who works on other sensory system look the intro over and underline the things that need to be explained to the unwashed.

>> We have edited the Introduction in an attempt to make it clearer and to reduce the use of jargon.

I know there are details in Nelson and Carney (2004), but it would help the reader if the parameters of the model were given in the caption of Fig. 2 (like the time delays, relative strength of E and I, time constants of the post-synaptic integration, and maybe the general structure of the integrate-and-fire (?) neurons. Also what parameter varies to move the modulation sensitivity around in Fig. 3.

-Good suggestion - All parameters are now provided in a Table. Note that as part of this effort, the parameters were stream-lined to make them as consistent as possible across figures, and all simulations were repeated with the final parameter values. Thus, there are small differences in all of the simulations from the original versions. But the qualitative results are the same (see page 8 of revised manuscript).

Results in Fig. 5 show a change in representation across levels. Can you speculate on constructing the ultimate neuron with a stable spectral representation at all levels?

-Vowels are intelligible across a wide range of levels; however, the perception of vowels changes with level, thus we're not sure there should be an 'ultimate' neuron that doesn't change with level. Indeed, as we point out, the representation in the model responses changes with level in an appropriate way (see below).

On the same subject, you state that the behavior at high sound levels in Fig. 5 is similar to the rollover in speech perception at high levels. It's not clear to me that there is a rollover in Fig. 5, certainly not in 5C and maybe not in 5B, so maybe the rollover would be more convincing in a postulated combo of the information in different modalities in Fig. 5? i.e. if you use rate-place information to code the spectra at low levels, where the modulation data are weaker, it might decrease the quality of the representation at high levels?

-We are proposing that the vowels are coded in the contrast in rates along the frequency axis. The contrast in the model response (i.e. the difference between peak and valley rates) is reduced at high levels (and it is strongest at the mid-levels). We have tried to clarify this point in the revised text (page 14 of revised text).

p 18, last section of Results: how many neurons in IC were studied and how many showed the modulation-dependent responses that improved spectral representation? From Nelson and Carney, it looks like about 47% of IC neurons are bandpass and 10% are band-reject. That means there are a lot of IC neurons for which the proposed mechanism is inappropriate. Although there is a brief admission of the issue of other MTF shapes in the discussion, there is not (1) a statement of the fraction of IC neurons that would behave as postulated here; (2) a statement that the neurons following the model proposed here are better than other IC neurons; and (3) a guess as to the role of this mechanism in the overall representation of speech in the IC. For example, how would the modulation-dependent neurons do at coding fricatives or stops? This is not to ask for more data, necessarily, but a couple of sentences in the Discussion hinting as to the big picture of IC spectral representation would be nice. Do you think there are multiple different spectral representations in IC? How does this one fit in?

-1) We have added a brief description of the percentages of neurons recorded that had responses that were significantly correlated to the model (page 20-21).

-2) It would be difficult to claim that the neurons consistent with the model are "better" than neurons for which we don't yet have good models. For example, the hybrid neurons are not well described by the two models suggested here, but they can be very sensitive to changes in modulation. More extensive testing is required to understand how the features of the hybrid neurons compare to others (e.g. "favorably" or "unfavorably", in terms of a robust representation across SPL and in noise). We don't presume that the cells that we are modeling are somehow "superior" in terms of coding speech sounds, just that we (may) understand the mechanisms governing their responses, and that they are robust across SPL and in noise.

-3) We have added a paragraph in the Discussion mentioning potential roles for these neurons for other speech sounds (page 26).

Maybe the journal doesn't allow this, but it would be helpful to have section titles ("model responses", "physiological responses", etc.) to give the reader a hint as to what is coming.

-We have added headings, as suggested.

Can you speculate on how the cochlear-implant simulations that simulate spectral-only signals with no fine structure would work in the perceptual model proposed? That is, we seem to be able to understand speech signals produced in this way. Presumably such stimuli would not generate the interaction of synchrony capture and modulation needed for the model presented here? I wonder how good vowel perception is for signals produced by the noise-vocoder models?

-CI simulations (i.e. vo-coded signals with no temporal fine structure) are not voiced, and thus this model would not apply to them. The same applies to other unvoiced vowels, such as whispered speech. In both cases, we presume that these sounds are coded not based on the required interaction of synchrony capture and modulation, but rather based on the energy profile in the spectrum. Vo-coded and whispered sounds are certainly not as clear as voiced vowels. How robust they are in the presence of noise is another question - we're not aware of any studies relevant to this question. We have added this issue to the Discussion.

SPECIFIC COMMENTS

p 9, l bottom - 3: "Author University animal care . . ." something's missing here (or are the authors at Author University?).

-This "University" is indicated by eNeuro's rules for anonymity in the initial submission. To be changed in final draft.

p 9, l bottom - 1: Bozeman, I think.

-Thanks - changed on pg. 11

p 9-10 spanning: were you studying single units? by what criteria?

-The criteria for single units have been added at the bottom of page. 11.

Fig. 4: it would help the reader to remind us in the caption that the vowel is at 65 dB SPL.

-Added.

p 15-16 and Fig. 7: can anything be said about the response maps of these neurons or other characteristics? (ITD sensitive, ILD sensitive, etc.). This is not very important.

-We have not seen any trends in these binaural properties and the MTFs; because this paper does not introduce any of the binaural properties. A description of binaural properties is beyond the scope of this study, but this is certainly a direction for future study.

More important is knowing what fraction of IC cells have BP and BR MTFs and behave in the way shown here?

-We have added a brief description of the numbers of cells that had responses that were statistically correlated to the model predictions. (Page 20-21).-

Second review - wants to re-review the revised Ms

This report represents the first attempt to relate the responses of midbrain neurons to complex (speech) stimuli. The advance in the approach is that the model is based on the known responses of peripheral neurons to the same stimuli, and the known nonlinearities in the response, and suggests a transformation that could account for the midbrain responses.

The paper posits a model for the responses of midbrain neurons, and based on the model, predicts responses to vowels. The paper goes further and compares the model outputs to responses of actual neurons, and shows that the model predictions roughly match the responses of neurons. This reviewer has some concerns, but overall, the manuscript has significant merit.

Major concern:

1. The entire paper is based on two example neurons. The authors must have more than just those two examples that convinced them that the model actually is a reasonable one for IC responses, so maybe some summary stats that show the goodness of prediction of more than one individual neuron of each type must be shown. And population plot of BP and LPBR neurons arranged by the unit BF would be a great match to Figures 4, 5, and 6.

-We have added 5 examples in a new Fig. 9, in addition to the original three examples, to provide the reader with a larger set of results. Unfortunately (see above), the limited number of cells in our population, and the considerable variability in response properties across IC cells, including discharge rates (many of which are non-monotonic), would make it very difficult to create the suggested population figure. We believe that a better strategy is to pursue recording of synthetic vowels, for which a single neuron can be studied more systematically that with the small set of vowels used in this initial (mainly) modeling study. These issues are now included in the Discussion (page 25).

2. The responses shown in Figure 8 are not internally consistent. The comparison of model with actual neuronal firing (Figure 8B) shows that the cell responds with a maximum at about 35 sp/s. However, the SPL series in Figure 8C for the same neuron (presumably) shows peak firing rates of 8 sp/s for 65 dB SPL, the conditions for B. Which is correct?

-Thanks for spotting this - there was an error in the vertical axis labels in one panel. The rates in Fig. 8C are now consistent with the other panels.

Minor:

1. The model section is great, and accounts for the responses of the two units shown (see major comment above). However, one goal of models is to account for other data as well. Can this model account for the differences in the frequency tuning and other basic response features between the IC and the auditory periphery (e.g. Borg et al. 1988) that have been demonstrated in other species such as the feline?

-We agree that models should be tested using different data than they were developed to explain, and this was in fact the case here. The model was developed to explain one of the major differences between monaural IC responses and peripheral responses: rate tuning to amplitude-modulation (AM) frequency. The model for AM-tuning (the SFIE model of Nelson &amp; Carney) was then applied to the vowel responses. The SFIE model does explain a few other basic properties of IC neurons, such as onset responses, phase-locking to AM, etc. (see Nelson &amp; Carney, 2004). However, many of the other basic properties of IC neurons are related to the binaural inputs of the vast majority of these cells, and this model does not attempt to account for any of these properties. Differences in frequency tuning between the model and IC responses were not investigated here; these responses are often affected by binaural interactions. These are certainly important issues for future studies.

I agree with the other reviewer about CN representations, specially the Chop S neurons (my bad about not remembering the Conley and Keilson paper, and the other papers including the ones by May and Sachs, describing Chopper neuron responses to vowels). The CN representations are critical to the model, so if the output of the CN node does not match the breadth of CN representation for the vowels, that at least merits a mention.

-Please see response to similar question from Reviewer#1.
